# Atomic Force Microscopy Study of the Arrangement and Mechanical Properties of Astrocytic Cytoskeleton in Growth Medium

**Published:** 2011

**Authors:** Yu.M. Efremov, E.V. Dzyubenko, D.V. Bagrov, G.V. Maksimov, S.I. Shram, K.V. Shaitan

**Affiliations:** Biological Department, Lomonosov Moscow State University; Institute of Molecular Genetics, Russian Academy of Sciences

**Keywords:** atomic force microscopy, dorsal root ganglia, force spectroscopy, confocal microscopy, cytoskeleton

## Abstract

Astrocytes are quite interesting to study because of their role in the development of various neurodegenerative disorders. The present work describes an examination of the arrangement and mechanical properties of cytoskeleton of living astrocytes using atomic force microscopy (AFM). The experiments were performed with an organotypic culture of dorsal root ganglia (DRG) obtained from a chicken embryo. The cells were cultivated on a gelatinous substrate and showed strong adhesion. AFM allows one to observe cytoskeleton fibers, which are interpreted as actin filaments and microtubules. This assumption is supported by confocal microscopy fluorescence imaging of α-tubulin and fibrillar actin. Mapping of the local Young’s modulus of a living astrocyte showed that the stiff areas correspond to the sites where the cytoskeleton fibers are located. Thus, the data obtained indicate that AFM is a promising method to study neural cells cytoskeleton integrity and arrangement in*in vitro*models of neurodegeneration.

## INTRODUCTION

Astrocytes are one of the main cell types in the central nervous system, where they have several functions: they direct and stimulate neuron migration during development; they sustain the neuronal microenvironment and modulate the immune response via antigen presentation [1]. The study of astrocyte morphology is of great interest due to their significant role in the pathogenesis of many common diseases of the central and peripheral nervous systems, such as ischemic stroke, Alzheimer’s disease, AIDS-related dementia [2], diabetic retinopathy [3], etc. These pathologies are accompanied by substantial morphological and physiological rearrangementsin neural cells and by changes in gene expression [2, 4]. They are also typically accompanied by changes in the cytoskeleton structure [5].

Atomic force microscopy (AFM) has been used in biological studies for a significant period of time for the visualization of biomolecules [6, 7] and cells [8–10] and for the assessment of their mechanical characteristics [11, 12]. AFM allows one to obtain a 3D image of a living cell in the growth medium and also perform manipulations on it at micro- and nano-scale. Cytoskeleton can usually be observed on cell images obtained in contact mode [9, 13]. Measuring the Young’s modulus makes it possible to get important information on the physiological and functional state of cells [14, 15]. In the current work, AFM has been used to study an organotypic culture of dorsal root ganglia (DRG) obtained from a chicken embryo. The astrocyte cytoskeleton structure has been examined, and the contribution of the cytoskeleton into the local Young’s modulus of a cell has been revealed. The data on the cytoskeleton arrangement obtained by AFM were compared with confocal microscopy data.

## EXPERIMENTAL

**Cell culture**

An organotypic culture of dorsal root ganglia (DRG) was obtained from a chicken embryo according to the standard procedure described elsewhere [16]. Briefly, to prepare gelatin-coated 35 mm Petri dishes, 2 ml of a 0.5% gelatin solution was placed into sterile dishes, incubated for 1 h at 37°С, and removed. Dorsal root ganglia were isolated from an 11- or 12-day-old chicken embryo under a binocular microscope and placed into the gelatin-coated Petri dishes. 2 ml of the F12 medium (Biolot, Russia) containing pyruvate, glutamine, penicillin, streptomycin and 10% of horse blood serum was added into each dish.

In order to enhance cell adhesion and prolong the lifetime of the primary culture, nerve growth factor (NGF 7S) was added at a final concentration of 5 ng/ml. The obtained samples appear as a mixed primary culture of neurons and astrocytes. These two cell types have clear morphological differences and therefore can easily be distinguished [1]. In order to detect astrocytes, immunocytochemical staining for glial fibrillary acidic protein (GFAP) (an astrocytic marker protein) was performed in accordance with a procedure described in [17].

**Atomic force microscopy**

The atomic force microscopy experiments were carried out on a Solver BIO atomic-force microscope (“NT-MDT”, Russia) equipped with a 100x100x7 µm ^3^ scanner and a closed-loop feedback system. The morphology intrinsic to living cells is likely to be retained for several hours, and the AFM experiments were performed only on the cells that had this type of morphology. The optical microscope, combined with the AFM, was used to choose the scanning region.

The measurements were performed in the culture medium using contact and semi-contact modes (the semi-contact mode did not help to improve the image quality; the information on the cytoskeleton was not available; therefore, we present only the images obtained in contact mode) using silicon nitride cantilevers MSCT-AUHW (former Veeco Instruments, now Bruker, USA). Trace and retrace topography and feedback error signal were recorded during each scan. The mechanical force acting on the cell, measured using the force-distance curves, was adjusted to be as small as possible (the typical value was equal to 1–4 nN) [18]. Comparison of the trace and retrace profiles was the criterion of correct feedback adjustment and validity of the obtained data. After the optimal scanning parameters were selected, the trace and retrace profiles showed good agreement, which indicated that there were no considerable distortions of the structure under the impact of the cantilever. The feedback error signal made it easier to reveal small surface relief heterogeneities [19]. The images were processed using ImageAnalysis (“NT-MDT”, Russia) and FemtoScan Online (Advanced Technologies Center, Russia) software.

In the force spectroscopy experiments, AFM was used to capture the force curves. A force curve is a plot showing the elastic force acting on the cantilever as a function of the vertical scanner displacement [20, 21]. To record a force curve, the cantilever is first pushed against the selected sample point. When the cantilever moves down, the approach curve is recorded, the maximum interaction force can be adjusted and is typically 2-3 nN. Then, the cantilever is lifted, providing the retract curve. Rectangular PNP-DB cantilevers (NanoWorld, Switzerland) and triangular MSCT-AUHW (former Veeco Instruments, now Bruker, USA) cantilevers made of silicon nitride were used for the force spectroscopy. Before the measurements, the rigidity of the rectangular cantilevers was determined using the Sader method [22, 23]; the rigidity values specified by the manufacturer were used for triangular cantilevers. The deflection was calibrated using the force curve obtained above the Petri dish surface. The Young’s modulus was calculated based on the approach curves using EF3 and the ImageAnalysis software (“NT-MDT”, Russia). In this software, the Sneddon’s modification of the Hertz model is used [9, 24].

**Confocal microscopy and fluorescent staining**

The specimens were fixed, stained with α-tubulin antibodies (DM1α, Santa Cruz Biotechnology, United States), and treated by the secondary antibodies conjugated with Alexa 594 (Alexa594 anti-mouse polyclone, Invitrogen, United States). Staining for astrocyte marker GFAP was performed in a similar way: after incubation with primary antibodies (GFAP, Abcam, England), secondary antibodies conjugated with Alexa 546 (Alexa546 anti-rabbit polyclone, Invitrogen, United States) were added. Rhodamine–phalloidin conjugate was used for actin staining. Fixation and staining were performed according to [17].

The confocal microscopy experiments were carried out using a LSM 510 META microscope (Carl Zeiss, Germany). Actin was visualised using an oil immersion objective lens Plan-Apochromat 100х “Carl Zeiss” (aperture 1.4), excitation wavelength 543 nm, spectral detection range 530–600 nm, and confocal diaphragm with 164 µm diameter. The image size was 1024 × 1024 pixels (85 nm/pixel).

Tubulin was visualised using an oil immersion objective lens Plan-Apochromat 100х “Carl Zeiss” (aperture 1.4), excitation wavelength 543 nm, spectral detection range 615–700 nm, and confocal diaphragm with 184 µm diameter. The image size was 1024 × 1024 pixels (85 nm/pixel).

GFAP was visualised using an oil immersion objective lens 63х “Carl Zeiss” (aperture 1.4), excitation wavelength 514 nm, spectral detection range 530–600 nm, and confocal diaphragm with 124 µm diameter. The image size was 1024 × 1024 pixels (127 nm/pixel).

## RESULTS AND DISCUSSION

**Visualization of astrocyte cytoskeleton using confocal and atomic force microscopy**

**Fig. 1:**
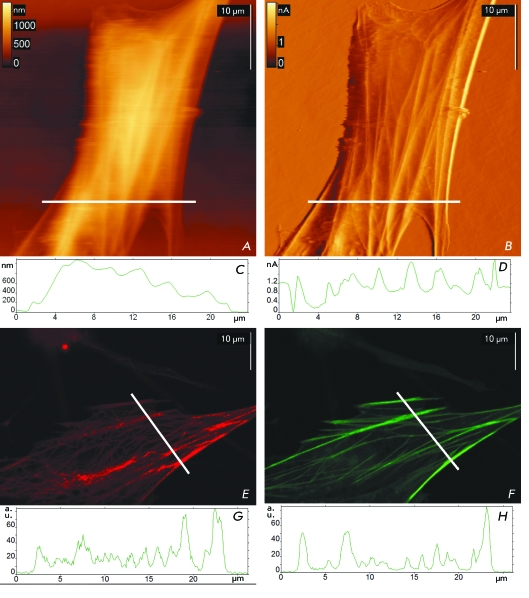
Visualization of the astrocyte cytoskeleton with AFM and confocal microscopy. Typical images are presented. *A* – the topographical image of the living astrocyte in growth medium. *B* – the corresponding contact error image. *C* – the height profile of the astrocyte measured with AFM along the white line on the topographical image. *D* – the contact error profile measured along the same line. *E* – immunocytochemical staining of α-tubulin microtubules (DM1A + Alexa594). *F* – staining of actin filaments with fluorescent phalloidin. *G,H* – profiles of the fluorescence intensity measured along the white lines on corresponding fluorescent images.

Studying living cells with AFM is a technically and methodically complex task, since living cells are very soft and thus are easily deformed by the cantilever; they require special conditions to keep viability and must be strongly bound to the substrate [10]. Accurate selection of the sample preparation method and elaborate adjustment of the scanning parameters are necessary to obtain reproducible results. 

Well-adhered cells cultured for 10 days were selected for scanning. The topographic images of living astrocytes obtained in contact mode show that the cells have an uneven surface with extended (fibrillar) structures ( *Fig. 1A* ). In contact mode, the cantilever pushes down the membrane and makes the submembrane cytoskeleton visible. It is most clearly observed on the feedback error signal images ( *Fig. 1B* ), which show the cantilever deflection in each point. The fibrillar structures are indiscernible in semi-contact mode. A similar result was obtained in the studies [8, 25].

The most clearly expressed and rigid intracellular structures are the actin and the microtubule networks [26]. We supposed that the structure of one or both of these networks could be visualized by scanning living astrocytes in contact mode in liquid.

To confirm this assumption, we compared the images obtained by AFM with those obtained by confocal microscopy upon immunofluorescence staining of astrocytes for α-tubulin and F-actin ( *Figs. 1E,F* ). The actin cytoskeleton, which is visible upon staining for F-actin, consists of long parallel fibrils ( *Fig. 1F* ). Staining for α-tubulin ( *Fig. 1E* ) provides the image showing the arrangement of microtubules in an astrocyte, which form a complex network. A similar network can also be seen on a topography image of a living astrocyte ( *Figs. 1A,B* ).

**Fig. 2:**
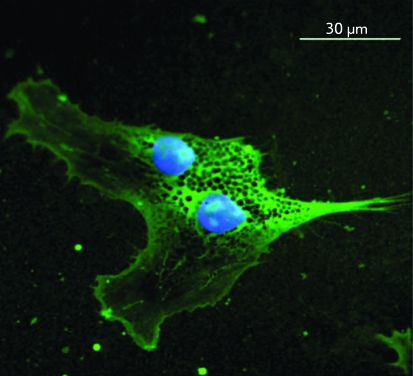
Immunocytochemical staining of a glial fibrillary acidic protein in the astrocytes of a DRG culture obtained from a chicken embryo. Green colour – Anti-GFAP + Alexa546, dark blue – nuclei stained with DAPI.

Intermediate filaments, which consist of GFAP in astrocytes, either do not form regular fibrillar structures in this culture or are destroyed upon immobilization ( *Fig. 2* ). Based on the comparison of the obtained images and on the basis of the published data [13, 27], we conclude that, unlike actin and microtubules, intermediate filaments in this case cannot be visualized by AFM.

The fluorescence intensity profiles were measured along the lines selected on the confocal microscopy images ( *Fig. 1G,H* ). When calculating the profile, the signal is averaged over several lines adjacent to the selected one. Thus, the presence of clearly discernible peaks on the profiles indicates the existence of extensive intracellular fibrils. Since fibrillar structures were detected in all three experiments ( *Figs. 1A,E,F* ), it cannot be claimed that the filaments observed by AFM in contact mode are necessarily microtubules or actin filaments. It is possible that both systems contribute to the formation of the surface topography ( *Fig. 1A* ); however, a number of researchers [13, 27] believe that it is actin cytoskeleton that plays the determining role.

It should also be noted that the peak width does not correspond to the diameter of an individual microtubule or actin filament in any of the measured profiles. It is well known [5] that microtubules are extensive α- and β- tubulin copolymers, 10 nm in diameter, whereas the diameter of an actin filament is 7–8 nm. Therefore, the visible structures are bundles of cytoskeleton components. Despite the complexity related to a clear differentiation of various cytoskeleton networks, its visualization by AFM has some advantage over immunocytochemical staining, since the AFM measurements can be performed on living cells in the culture medium. 

Thus, the astrocytes of an organotypic culture of dorsal root ganglia obtained from a chicken embryo cultivated on a gelatinous substrate are well adhered, do not shift during scanning, and possess a high level of viability. The gelatinous substrate can efficiently substitute the substrates made of polyornithine/laminine, collagen, etc., which are more expensive and require rather complicated preparations [28].

**Force spectroscopy and measurement of the local Young’s modulus of living astrocytes**

**Fig. 3:**
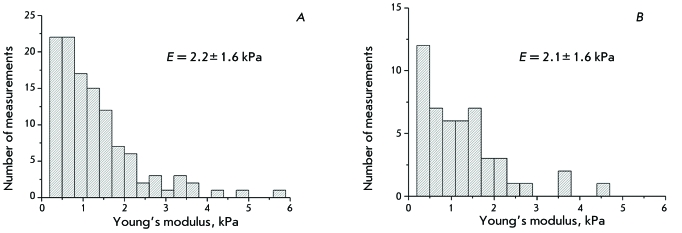
Histograms of the astrocyte Young’s modulus. *A* – obtained with long cantilever PNP-DB, k = 0.02 N/m. *B* – obtained with short cantilever PNP-DB, k = 0.18 N/m.

To get more information about the cytoskeleton, we measured the local Young’s modulus of the living astrocytes from the force spectroscopy data. The force curves were recorded in the points located along the selected lines (10–20 points per line) or on the grid (from 4 × 4 to 7 × 7 points). Measurements were carried out using two cantilevers (their rigidity values differed by an order of magnitude and were equal to *k*_1 _ = 0.02 N/m and  *k*_2 _ = 0.18 N/m) in order to demonstrate that the method chosen to calculate the Young’s modulus is valid. The values of the Young’s modulus of two or three cells were measured using each cantilever. The obtained histograms matched well ( *Fig. 3* ), and so did the average values of the Young’s modulus *Е*_1 _ = 2.2 ± 1.6 kPa and  *Е*_2 _ = 2.1 ± 1.6 kPa. This proves that the performed measurements are valid. The Young’s modulus values determined on living astrocytes fall into a wide range (0.36–9.6 kPa), which is typical of eukaryotic cells. It is known that the Young’s modulus values vary from 0.02 to 400 kPa in different eukaryotic cells [14] (the range is from 1 to 40 kPa for astrocytes obtained from rat cerebrum [13]). It is also known that the average Young’s modulus of culture-dissociated DRG neurons adhered on polyornithine/laminin [29] is equal to 60 kPa. This fact correlates with the data [11, 30] that the astrocytes are softer than the neurons.

**Fig. 4:**
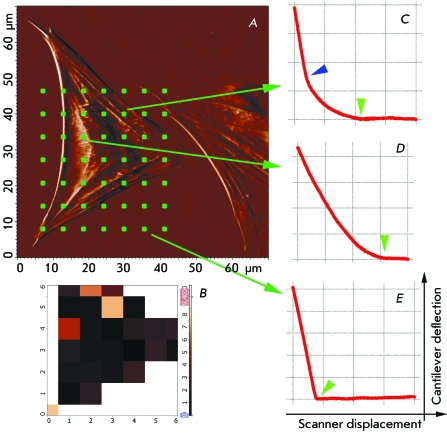
Mapping the local Young’s modulus of the astrocyte. *A* – the deflection image of the living astrocyte and a grid of points where the force curves were obtained. *B* – the map of the local Young’s modulus in the grid nodes. The colour scale is in kPa, lighter squares correspond to stiffer areas. *C* – the force curve obtained in a point above the cell edge, the upper part of the curve coincides with the curve obtained on the substrate (E). Green triangular pointers mark the contact point, and blue triangular pointer marks the point where the cantilever touches the substrate. *D* – the force curve obtained in a point above the cell nucleus. The range of the scanner displacement on all curves was 2 µm.

**Fig. 5:**
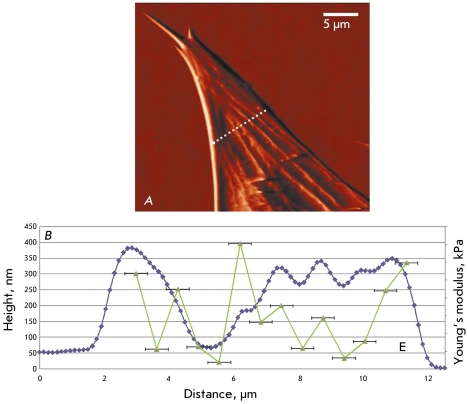
Mapping the local Young’s modulus of the astrocyte. *A* – the image of the astrocyte and the line along which the force curves (14 points) were obtained. *B* – the height profile along the section line (the dark blue curve) and the values of the local Young’s modulus (the green points) measured in the corresponding points. Horizontal error bars show the size of the contact area. In the areas where the cytoskeleton fibers are located (local maxima on the dark blue curve), the local Young’s modulus increases.

Mapping of the Young’s modulus was also performed; the force curves were recorded in the points located on the grid nodes ( *Fig. 4* ). On the elasticity map, the lighter squares correspond to the areas with higher rigidity, whereas the darker squares correspond to the areas with lower rigidity ( *Fig. 4B* ). The force curves are also shown in different points ( *Fig. 4C,D,E* ). The curve has an abrupt slope above the substrate; above the edge of the cell, the curve is smooth until the cantilever interacts with the substrate. The curves are smooth above the nucleus and more abrupt above the cytoskeleton fibrils. When the force curves along the chosen line were recorded, it was observed that the curve shape and the Young’s modulus depended on the presence of the cytoskeleton below the membrane. If there were elements of the cytoskeleton at the reference points (they could be seen on the topographic images), the calculated Young’s modulus was higher ( *Fig. 5* ), which supports the data [13]. It should be mentioned that when a force curve is recorded, the Young’s modulus is averaged over the size of the contact area between the cantilever tip and the cell surface. The size of the contact area depends on the probe geometry and indentation depth; in this experiment, it was approximately equal to 700 × 700 nm [31]. This fact can be accounted for by the scattering in the values of the Young’s modulus measured above the cytoskeleton elements. Moreover, the fibrils could correspond to the bundles of cytoskeleton filaments of different densities, which impacts the local rigidity. It is obvious from the diagrams of the Young’s modulus values ( *Fig. 3* ) that the majority of points fall into the regions of the cell surface where there are no cytoskeleton elements.

## CONCLUSIONS

The obtained data demonstrate that the morphology of astrocytes in an organotypic culture of chicken embryo DRG grown on a gelatinous substrate can be successfully studied by AFM. The cells prepared in this manner are well-adhered, viable, and do not shift substantially during scanning, which makes it possible to use gelatin as an inexpensive and reliable substrate for this culture. The high resolution of the AFM method allows one to observe the cytoskeleton arrangement of a living cell in the culture medium. Unlike confocal microscopy, AFM does not provide information to determine which of the cytoskeleton networks is observed. However, AFM is promising for the study of the cytoskeleton organization in experiments on living cells. Moreover, since the local Young’s modulus of a cell is considerably higher at the sites of cytoskeleton fibrils location, force spectroscopy allows one to determine cytoskeleton integrity and rearrangement upon damage. The study of the changes in the cytoskeleton integrity of neuronal cells upon neurodegenerative conditions appears to be a prospective application of AFM. When studying cytoskeleton degradation (one of the key processes in the development of neurodegeneration [32]), force spectroscopy will facilitate a quick, non-invasive and accurate determination of the Young’s modulus of living neuronal cells. 

## Abbreviations

AFM: atomic force microscopy
DRG: dorsal root ganglia
GFAP: glial fibrillary acidic protein
